# Gamification design and engagement in preregistration nurse education: a scoping review protocol

**DOI:** 10.1136/bmjopen-2025-107037

**Published:** 2026-01-12

**Authors:** Kelvin McMillan, Tracey Valler, Amelia Swift

**Affiliations:** 1 Department of Nursing and Midwifery, University of Birmingham, Birmingham, UK; 2 Nursing, University of Birmingham, Birmingham, UK

**Keywords:** Health Education, Nurses, Education, Medical

## Abstract

**Abstract:**

**Introduction:**

The complexity of modern healthcare has driven an increase in the complexity of the preregistration nursing curricula. Diverse learning needs in this population are best served by inclusion of diverse approaches to teaching. Gamification offers an approach to enhance motivation and engagement, allowing for sustained motivation to keep learning. However, current research concerning gamification within preregistration nursing is still limited, particularly surrounding underlying design and the impact this has on long-term engagement and motivation. The aim of this scoping review is to identify and map gamification design elements used in preregistration nursing education, using the Octalysis framework, and to evaluate how these designs influence student engagement and motivation.

**Methods and analysis:**

This scoping review will use the updated Joanna Briggs Institute Scoping Review Methodology and will be reported in accordance with Preferred Reporting Items for Systematic Reviews and Meta-Analyses extension for scoping reviews (PRISMA - ScR). The search will be conducted using Medical Literature Analysis and Retrieval System Online (MEDLINE), Cumulative Index to Nursing and Allied Health Literature (CINAHL), Education Resources Information Cente (ERIC), EBSCO, Web of Science Core Collection, PROquest, SCOPUS, Excerpta Medica Database (EMBASE) and PsycINFO. Grey literature, conference proceedings and relevant digital platforms will also be considered. Two reviewers will independently screen titles/abstracts and full texts. Data extraction will include gamification design elements, engagement and motivation outcomes and their alignment with the Octalysis framework. Synthesis and presentation of findings will be completed using the Patterns, Advances, Gaps, Evidence for practice, Research recommendations framework. The planned start for performing the scoping review is November 2025.

**Ethics and dissemination:**

Ethical approval is not required as this review will synthesise published and publicly available evidence. Findings will be disseminated via peer-reviewed publication, conference presentations and stakeholder engagement within higher education.

**STRENGTHS AND LIMITATIONS OF THIS STUDY**

- This review will map gamification design elements in preregistration nursing education to the Octalysis framework and collate reported effects on engagement and motivation.
- A comprehensive search of published and unpublished literature will be conducted across multiple databases and grey literature sources.
- The synthesis will use the Patterns, Advances, Gaps, Evidence for practice, Research recommendations framework to identify patterns, advances, gaps and recommendations.
- Inclusion of non-English language papers will allow more comprehensive exploration.
- No formal quality appraisal will be conducted, consistent with scoping review methodology.

## Introduction

 Nursing students entering today’s healthcare environment face increasingly complex clinical challenges due to multimorbidity, rapid technological advances, complex and pharmacological advances and increasing life expectancy.^1^ This necessitates a more complex curriculum to prepare nurses to work safely and effectively. Preparing nursing students for this level of practice requires educational approaches that cultivate empathy, adaptability, critical thinking and interpersonal communication.^2 3^ Therefore, engaging nursing students meaningfully in their learning is not only an academic goal, but a professional imperative aligning with the evolving challenges within healthcare settings.

Teaching and learning methods constantly evolve in higher education, and questions are asked about whether traditional teaching methods resonate with diverse learning preferences.^4^ Modern learners, particularly those recognised as ‘Generation Z’, have become accustomed to technology-rich environments, showing preference to personalised and interactive learning that is supported by instant feedback.^5 6^ Furthermore, there is diversity within the nursing student population in terms of age, ethnicity, life experience and educational backgrounds.^7^ Reliance on didactic teaching approaches is insufficient to maintain adequate and sustained engagement in learning.

Engagement is defined as the behavioural, emotional and cognitive investment students make in their learning.^8 9^ This links closely to motivation, as motivation is needed to trigger this investment. Motivation is shaped by a range of factors including intrinsic interest, perceived relevance and personal value of the learning content.^10^ For meaningful engagement to be achieved, learners need to feel a sense of relevance towards the learning goals, therefore feeling motivated to sustain attention and immerse themselves within learning environments.^11^ Didactic learning approaches can limit meaningful engagement.^12^

Gamification is something that offers a way to enhance engagement by creating flexible, inclusive and dynamic learning environments. Gamification is a pedagogical approach used to integrate game mechanics into non-game contexts. It involves the inclusion of game mechanics traditionally used within game design to enhance engagement, motivation and learning outcomes.^13^ It draws on theoretical foundations such as constructivism and active learning, both of which encourage cognitive processing, learning through experiences and being actively involved in challenging situations.^14^ The emphasis is on the learner having a key role in creating their own learning opportunities.

Within nursing education, gamification has been trialled through approaches such as escape rooms, serious games, simulation-based activities and virtual or augmented reality. Reported benefits include increased learner satisfaction, enhanced problem-solving abilities and higher levels of participation.^15 16^ However, while gamification strategies are being adopted across nursing curricula, there is still limited clarity on which gamification designs best support engagement and motivation, and why certain strategies are more effective than others.^17^

Published reviews have begun to explore gamification within health education more broadly. Between 2012 and 2024 there were 475 papers published, with an increase in recent years and 101 publications in 2023.^18^ These reviews have highlighted growing interest in gamification, but there is limited focus on preregistration nursing curricula. Many reviews^19^,^24^ found that the effects of gamification in nursing education improve knowledge acquisition, learner satisfaction and short-term motivation. Seo *et al*^25^ conducted a systematic review and meta-analysis exploring the effects of gamification on nursing students’ motivation and learning outcomes, concluding that gamification can positively influence learning motivation and performance. A similar review^26^ has focused on quantifying educational outcomes or describing general gamification strategies used in nursing curricula.

However, despite this growing body of evidence, the design mechanisms underpinning gamification remain underexplored. Existing reviews primarily focus on outcomes in what gamification achieves, rather than how specific design features or motivational drivers influence engagement and motivation. Furthermore, no published review has systematically mapped gamification design elements within preregistration nursing education against a comprehensive motivational framework. A small number of recently registered protocols suggest continuing interest in these topics, yet none explicitly apply a design-focused analytical framework. This highlights a persistent gap in the literature, where understanding the relationship between gamification design, motivational theory and learner engagement is essential for guiding effective and theory-informed curriculum design in nursing education.

To ensure that no similar scoping review is currently being conducted, searches were performed in the Open Science Framework (OSF) and PROSPERO protocol registries. One OSF protocol registered in 2023^27^ proposed a systematic review and meta-analysis examining the influence of gamification in formal education settings on learner motivation. However, the protocol appears to be incomplete and has not been updated since registration. Another OSF protocol registered in 2024^28^ aims to review gamification and serious games in nurse education, focusing on the implementation challenges of serious games rather than engagement or motivation. This indicates no substantive overlap with the present review.

In PROSPERO, an ongoing systematic review was identified exploring gamification in palliative care education.^29^ This protocol specifies neither motivation nor engagement as outcomes of interest. Ma *et al*^30^ have also registered a review focusing on medication-safety education for nursing students, which does include motivation and engagement outcomes but is restricted to that single educational topic. Collectively, these registered protocols demonstrate growing scholarly interest in gamification but confirm that no existing or planned review has specifically mapped gamification design in preregistration nursing education using a theoretical framework.

To address this gap, this review will apply the Octalysis framework,^31^ a comprehensive model of human motivation that identifies eight core drives: (1) epic meaning and calling; (2) development and accomplishment; (3) empowerment of creativity and feedback; (4) ownership and possession; (5) social influence and relatedness; (6) scarcity and impatience; (7) unpredictability and curiosity and (8) loss and avoidance. Together, the core drives capture intrinsic and extrinsic sources of motivation that influence how learners engage with gamified environments. While other models commonly used in education, such as Self-Determination Theory,^32^ or Flow Theory,^33^ offer valuable insights, they focus on specific dimensions of motivation. The Octalysis framework provides a broader, integrative lens that allows for systematic mapping of gamification design to motivational drivers. This makes the Octalysis framework suitable for this review.

By collating evidence across diverse gamification interventions and mapping them against the Octalysis core drives, this review will provide a structured overview of how gamification is being designed, and the extent to which it influences student engagement and motivation. This will help identify gaps in the evidence base, which can guide future curriculum development and research in nursing education.

### Aim

The aim of this scoping review is to identify gamification design elements used in preregistration nursing education, map them to the Octalysis core drives, and evaluate their reported impact on student engagement and motivation.

The review will address the following objectives:

1. What gamification design elements have been used in preregistration nursing education?
2. Which Octalysis core drives do design elements align with?
3. What reported effects on student engagement and motivation are associated with those design elements?
4. What is the evidence gap and recommendations for future gamification design and research?

## Methods

### Design

The proposed scoping review will be conducted using the updated Joanna Briggs Institute (JBI) methodology for scoping reviews.^34^ The review will be reported using the Preferred Reporting Items for Systematic Reviews and Meta-Analyses extension for scoping reviews checklist.^35^ The protocol has been registered with the OSF to enhance transparency and reduce risk of duplication.

### Eligibility criteria

Eligibility will be guided by the Participants, Concept and Context framework:

### Inclusion criteria

#### Participants

Preregistration nursing students.

#### Concept

Studies investigating gamification as a pedagogical approach to support teaching and learning within preregistration nursing education, both digital and non-digital applications. Gamification design elements will be mapped to the Octalysis framework and evaluated in terms of their reported effects on engagement and motivation.

#### Context

Preregistration nursing education within higher education institutions.

#### Study designs

Primary research studies (quantitative, qualitative or mixed methods), literature reviews, theses, conference proceedings and other grey literature reporting empirical data. Inclusion of studies was determined by the preliminary search in PubMed to explore types of literature that provides engagement data and gamification strategies with the aim to increase engagement.

#### Publication characteristics

Publications from 2010 onwards, as this year is regarded as a defining moment in the history of gamification with a TED (Technology, Entertainment, Design) Talk by Dr Jane McGonigal, titled Gaming Can Make a Better World.^36^ All languages will be considered, and non-English publications will be translated by independent members of staff or using translation software. Use of AI (Artificial intelligence) will be transparent and reviewed by all authors to ensure accuracy.

### Exclusion criteria

- Studies involving post-registration nurses, continuing professional development or interprofessional cohorts where nursing student specific data cannot be separated.
- Studies that use serious games or simulation-based learning without integration of gamification elements.
- Editorials, opinion pieces and purely theoretical papers without primary or secondary data.

### Information sources and search strategy

An initial search strategy was conducted via PubMed, and CINAHL to generate search terms. Analysis of text words contained within identified articles has helped with the development of a full search strategy. This search strategy will be performed on the identified databases: MEDLINE (PubMed), CINAHL (EBSCO), ERIC, Web of Science Core Collection, PROquest Central, SCOPUS, EMBASE and PsycINFO. Each identified key term will be adapted for each database planned. All sources of evidence will have reference lists screened to identify suitable studies and literature that address the research question. The search strategy was reviewed by two review authors. The full search strategy conducted in October 2025 in MEDLINE and CINAHL can be found in table 1.

**Table 1:** Full search strategy conducted October 2025

	Search strategy stages	PubMed results	CINAHL results
S1	Gamification (Mesh))OR gamification* OR gamif* OR game-based* OR serious game* OR game* OR game-like* OR serious play OR Video Games (Mesh))video game* OR gamified learning OR game element* OR digital game OR Games, Experimental (Mesh))OR experimental game* OR game dynamic*	128 984	35 136
S2	Students, Nursing (Mesh))OR Nursing student* OR student nurse* OR preregistration nurs* OR undergraduate nurs* OR Nursing Education (Mesh))OR nursing education	291 845	160 196
S3	learn* OR engag* OR participat* OR motivat* OR active learning	2 341 531	743 313
S4	S2 AND S3	78 301	45 545
S5	S1 AND S4	1194	772
S6	S3 AND S8 FILTER: Full Text	1123	25
S7	S3 AND S8 FILTERS: Full Text and From 2010	998	21

To maximise coverage, grey literature (including conference proceedings, theses, institutional repositories, digital gamification platforms) will also be searched. Grey literature is included to capture innovative applications of gamification that may not yet be published in peer-reviewed journals. This ensures a more comprehensive mapping of strategies.

### Study selection

The citations and abstracts will be transferred to Covidence Systematic Review software^37^ for de-duplication and screening. A pilot test of the screening criteria will be conducted on a random sample of 25 titles and abstracts by 2 independent reviewers, after which the team will discuss discrepancies and refine the eligibility criteria. Full screening will begin when at least 75% agreement is achieved among reviewers. Full-text articles will then be reviewed and reasons for exclusion will be documented. Any disagreements between the reviewers at any stage of the review will be resolved through discussion, and if necessary by discussion with an additional reviewer.

### Data charting and extraction

Data will be extracted by two independent reviewers in Covidence. The data extraction tool will be piloted by two independent reviewers. The data extracted will include details of gamification design elements, associated Octalysis core drives and reported outcomes relating to engagement and motivation (table 2).

**Table 2:** Data extraction form draft

Category	Type of data
Bibliographic information	Author
	Year of publication
	Country of origin
	Aims/purpose of the study
Research questions	Subject
	Methodology
	Gamification strategy
	Outcomes and details of these
	Key findings (alignment with Octalysis framework)

The data extraction tool will be modified and revised as necessary during the process of extracting data from each included evidence source. Modifications will be detailed in the report. Any disagreements that arise between the reviewers will be resolved through discussion, or with an additional reviewer(s). If appropriate, authors of papers will be contacted to request missing or additional data, where required.

### Data synthesis and presentation

Narrative synthesis will be used to analyse and present findings. To support this, the review will use the Patterns, Advances, Gaps, Evidence for practice, Research recommendations framework.^38^ This approach supports systematic mapping of gamification design elements to the Octalysis core drives and facilitates identification of underexplored areas. Descriptive statistics will also be used to summarise study characteristics.

## Discussion

The objective of this review is to describe the range of gamification design strategies in preregistration nursing education, to map them against the Octalysis core drives, and to evaluate their reported impact on student engagement and motivation. Performing a scoping review allows for a broad range of literature to be extracted, helping to align gamification strategies to the core drives within the Octalysis framework. The review will provide a reproducible search strategy that aligns to the updated JBI methodology for scoping reviews. The review will not analyse the quality of research undertaken within extracted literature, more focusing on identification of gamification strategies, engagement results and evaluation methods. The review will offer nursing educators and other associated professionals’ crucial findings on which key motivational drives should be investigated in future research, while highlighting key motivational drives that are optimising nursing student’s experience with gamification.

### Ethics and dissemination

No ethical approval was required due to data consisting of published studies and findings. No primary research involving individual data from human participants was performed. Dissemination will include publication in a peer-reviewed journal, presentation at conferences and presenting findings to stakeholders with a shared interest in this area of research.

### Patient and public involvement statement

No patient and public involvement in the provided protocol.

## Data Availability

No data are available.
