# Supplementary material for: Gamification design and engagement in preregistration nurse education: a scoping review protocol
Source: BMJ Open. 2026 Jan 12;16(1):e107037. doi: 10.1136/bmjopen-2025-107037 (PMC12815113; doi:10.1136/bmjopen-2025-107037)
Supplement: Supplementary file 1 [file bmjopen-2025-107037.reviewer_comments.pdf]

PEER REVIEW HISTORY

BMJ Open publishes all reviews undertaken for accepted manuscripts. Reviewers are asked to complete a checklist review form and are provided with free text boxes to elaborate on their assessment. These free text comments are reproduced below.

ARTICLE DETAILS

Title (Provisional)

Gamification Design and Engagement in pre-registration nurse education: A Scoping Review Protocol

Authors

McMillan, Kelvin; Valler, Tracey; Swift, Amelia

VERSION 1 - REVIEW

**Reviewer**	**1**
**Name**	**Kong, Ling-Na**
**Affiliation**	**Chongqing Medical University, School of Nursing**
**Date**	**04-Sep-2025**
**COI**	**None**

General comments

-The title was the "core drives influence gamification design", however, the objective of this review was the "gamification design and impact on engagement and motivation". This was inconsistent. What is the main objective of this review? Please make clear on it and revise it throughout the manuscript.

-Revise the structure of the manuscript based on other published scoping review articles in the journal of BMJ Open.

- Please give the registration information on this review.

Abstract

-Change "Background" to "Instruction".

- Please reconsider the key words. Not including "pre-registration" and "engagement"?

-Page 3 Line 21 Please clarify impact of gamification on motivation? or engagement ? or motivation/engagement? And keep it consistent throughout the manuscript.

Background

-Page 3 Line 32 Change "Background" into "Introduction".

-In this section, the authors did not give enough information about this topic, for example, existing studies on application of gamification in the pre-registration nursing curricula. Therefore, the reasons of this review were not clear, which weakened the importance and necessity of this review. Strongly suggest to give more information on the background in this section.

- Suggest to make simple descriptions on the content of Table 1 in the text, not using a table.

- Page 4 Line 41 – Page 5 Line 10 Why described the eight theories? Are these theories closely related to this review?

- This section needs reasonable modifications.

Review Questions

-Page 6 Line 37 Please give full name of PCC?

-Just provide questions, not provide inclusion criteria in this section.

Methods

- Eligibility Criteria is unclear. Make detailed information on each criterion, including both inclusion and exclusion criteria.

-Inclusion criteria: suggest to describe this section from participants, setting, study design, content, etc.

- The authors should provide clear and detailed search strategy for each database mentioned in the Search strategy section.

- Why include "grey literature"?

- Make more detailed description on the section of "Analysis of evidence".

- Add the timelines.

**Reviewer**	**2**
**Name**	**Paiva-Santos, Filipe**
**Affiliation**	**Higher School of Nursing of Coimbra Health Sciences Research Unit Nursing, Fundamental Nursing**
**Date**	**07-Sep-2025**
**COI**	**None**

Congratulations to the authors on the submission of protocol "What core drives influence gamification design in pre-registration nurse education?". This is a timely manuscript that addresses a highly relevant and emerging area in nursing education, with the potential to

inform future pedagogical strategies and enhance student engagement through evidence-based gamification design.

This manuscript presents a scoping review protocol, developed in accordance with JBI guidelines, aiming to explore and present key gamification design used within pre-registration nursing education and evaluate the impact on engagement and motivation using the Octalysis Framework.

The manuscript includes 28 references, of which 18 (~ 64%) were published within the last five years. The references are relevant, encompassing key areas such as gamification, nursing education, student engagement, and motivational theory.

Title

Appropriate and include the term "scoping review protocol".

Abstract

Taking into account that this is a scoping review protocol, I suggest structuring with background, methods and conclusion.

Keywords

All terms are MeSH and appropriate.

Background

The authors could better frame gamification: what are the advantages of gamification in learning, what studies already exist on gamification in nursing education.

The justification for the need for this scoping review would benefit from a more explicit articulation of the specific gap in the literature. While the authors state that the impact of gamification design on engagement remains underexplored, they do not critically examine existing reviews or clearly demonstrate what has not yet been addressed. The choice of the Octalysis Framework, although explained, would be stronger if supported by a comparative analysis with other motivational models commonly used in nursing education.

The authors have chosen to include only English-language publications, which is understandable for practical reasons; however, this approach may inadvertently exclude valuable scientific literature published in other widely used academic languages such as Spanish or Portuguese. Given the increasing availability of reliable translation tools, including those powered by artificial intelligence, the inclusion of non-English sources could enrich the review and strengthen its global relevance.

Review question

I suggest that the authors present the research question(s) in this section (in the form of a question), and then start another section with the eligibility criteria (participants, concept and context, as well as the types of sources). I suggest that authors strictly follow the structure of JBI's scope review protocols.

The authors use the mnemonic PCC. The concept and context have a confusing description.

Methods

The authors do not refer to whether they have done a previous search in a review protocol registration repository (such as the OSF),To ensure that no one is doing a scope review like the one presented in this manuscript, and also do not refer to whether they registered the protocol. Although protocol registration is not mandatory, it is highly recommended to avoid duplication of revisions.

Search strategy

I suggest correcting some errors like those presented in S1: if one of the terms is "gamif*" it is not necessary to have the term "gamified learning"; if one of the terms is "game" the terms "game elements" or "game mechanics" or "game design" or "eduactional game" are not necessary.

Data extraction

In table 3 the authors report that they will extract data on the population of the studies included in the scope review. Does it make sense? Won't they always be undergraduate nursing students?

Analysis of evidence (suggested to replace with data analysis and presentation)

How will the authors relate the extracted data to the Octalysis framework?

VERSION 1 - AUTHOR RESPONSE

Comment	Action and rationale
Title	
Appropriate and include the term "scoping review protocol".	Title is now: Gamification Design and Engagement in pre-registration nurse education: A Scoping Review Protocol
Abstract	
Change "Background" to "Introduction".	Subheading changed to Introduction
Taking into account that this is a scoping review protocol, I suggest structuring with background, methods and conclusion	Abstract structure revised as advised.
Please reconsider the key words. Not including "pre-registration" and "engagement"	Key words have been amended as advised.
All terms are MeSH and appropriate.	No changes made
Page 3 Line 21 Please clarify impact of gamification on motivation? or	Further emphasis has been made on the impact of gamification on motivation and

engagement ? or motivation/engagement? And keep it consistent throughout the manuscript	engagement. Consistency has been maintained throughout the manuscript.
Background	
Page 3 Line 32 Change "Background" into "Introduction".	Subheading changed.
In this section, the authors did not give enough information about this topic, for example, existing studies on application of gamification in the pre-registration nursing curricula. Therefore, the reasons of this review were not clear, which weakened the importance and necessity of this review. Strongly suggest to give more information on the background in this section.<br><br>The justification for the need for this scoping review would benefit from a more explicit articulation of the specific gap in the literature. While the authors state that the impact of gamification design on engagement remains underexplored, they do not critically examine existing reviews or clearly demonstrate what has not yet been addressed.	Additional paragraph added to introduction to include recent, existing studies on the application of gamification in pre-registration nursing curricula. This has allowed for critical justification for the need of the scoping review.
The authors could better frame gamification: what are the advantages of gamification in learning, what studies already exist on gamification in nursing education.	Additional information has been added that explains the advantages of gamification, and the growing interest in gamification within healthcare education.
Suggest to make simple descriptions on the content of Table 1 in the text, not using a table	Table 1 has been removed and simple descriptions included.
The choice of the Octalysis Framework, although explained, would be stronger if supported by a comparative analysis with other motivational models commonly used in nursing education.	Comparative analysis between the use of Self-Determination Theory and Flow Theory made to strengthen rationale for the use of the Octalysis Framework.
The authors have chosen to include only English-language publications, which is understandable for practical reasons; however, this approach may inadvertently exclude valuable scientific literature published in other widely used academic	Additional information has been added to include all international literature, as the use of AI to assist with translation is an option. Use of AI will be clearly explained in the final scoping review methods.

languages such as Spanish or Portuguese.	
Page 4 Line 41 – Page 5 Line 10 Why described the eight theories? Are these theories closely related to this review	The idea was to show how the theories are related to the Octalysis Framework. These have since been removed.
This section needs reasonable modifications	
Review Questions	
Page 6 Line 37 Please give full name of PCC<br><br>The authors use the mnemonic PCC. The concept and context have a confusing description.	This has been altered
Just provide questions, not provide inclusion criteria in this section.	This has been completed.
I suggest that the authors present the research question(s) in this section (in the form of a question), and then start another section with the eligibility criteria (participants, concept and context, as well as the types of sources).	This has been altered following advice.
I suggest that authors strictly follow the structure of JBI's scope review protocols.	The scoping review protocol now strictly follows the JBI's scoping review protocol guidance.
Methods	
Eligibility Criteria is unclear. Make detailed information on each criterion, including both inclusion and exclusion criteria.	This has been amended.
Inclusion criteria: suggest to describe this section from participants, setting, study design, content, etc.	This has been amended
The authors should provide clear and detailed search strategy for each database mentioned in the Search strategy section	This will be included as an appendix.
Why include "grey literature"	Justification has been strengthened.
Make more detailed description on the section of "Analysis of evidence".	More detail has been added.
Add the timelines	This has been added.
Given the increasing availability of reliable translation tools, including those powered by artificial intelligence, the inclusion of non-English sources could	Additional information has been added to include all international literature, as the use of AI to assist with translation is an option. Use of AI will be clearly explained in the final scoping review methods.

enrich the review and strengthen its global relevance.	
The authors do not refer to whether they have done a previous search in a review protocol registration repository (such as the OSF),To ensure that no one is doing a scope review like the one presented in this manuscript, and also do not refer to whether they registered the protocol. Although protocol registration is not mandatory, it is highly recommended to avoid duplication of revisions.	This has been added and registration to OSF has been completed.
I suggest correcting some errors like those presented in S1: if one of the terms is "gamif*" it is not necessary to have the term "gamified learning"; if one of the terms is "game" the terms "game elements" or "game mechanics" or "game design" or "eduactional game" are not necessary.	This has been altered and will be provided as a supplementary file
In table 3 the authors report that they will extract data on the population of the studies included in the scope review. Does it make sense? Won't they always be undergraduate nursing students?	This has been altered within the supplementary file.
Analysis of evidence (suggested to replace with data analysis and presentation)<br>How will the authors relate the extracted data to the Octalysis framework?	This has been amended with a clear link to the Octalysis Framework.
